# Reappraising the relationship between hyperinsulinemia and insulin resistance in PCOS

**DOI:** 10.1530/JOE-24-0269

**Published:** 2025-03-12

**Authors:** Emma J Houston, Nicole M Templeman

**Affiliations:** Department of Biology, University of Victoria, Victoria, British Columbia, Canada

**Keywords:** polycystic ovary syndrome, insulin, androgens, metabolic dysfunction, insulin hypersecretion, insulin clearance, insulin sensitivity

## Abstract

Polycystic ovary syndrome (PCOS), a reproductive endocrine disorder with quintessential features of metabolic dysfunction, affects millions of women worldwide. Hyperinsulinemia (i.e., elevated insulin without hypoglycemia) is a common metabolic feature of PCOS that worsens its reproductive symptoms by exacerbating pituitary hormone imbalances and increasing levels of bioactive androgens. Hyperinsulinemia in PCOS is often attributed to insulin resistance, based on the concept that impaired insulin-mediated glucose disposal would induce compensatory insulin hypersecretion. However, it is challenging to define the sequential relationship between insulin sensitivity and insulin secretion, as they are tightly interlinked, and evidence suggests that hyperinsulinemia can alternatively precede insulin resistance. Notably, other drivers of hyperinsulinemia (outside of insulin resistance) may be highly relevant in the context of PCOS. For instance, high androgen levels can augment both hyperinsulinemia and insulin resistance, generating a self-perpetuating cycle of reproductive and metabolic dysfunction. In this review, we evaluate the cause-and-effect relationships between insulin resistance and hyperinsulinemia in PCOS. We examine evidence for the prevailing theory of insulin resistance as the primary defect that causes secondary compensatory hyperinsulinemia, and an alternative framework of hyperinsulinemia as the earlier defect that perpetuates reproductive and metabolic features of PCOS. Considering the heterogeneous nature of PCOS, it is improbable that its metabolic characteristics always follow the same progression. Comprehensively examining all mechanistic regulators of hyperinsulinemia and insulin resistance in PCOS might thereby lead to improved prevention and management strategies, and address critical knowledge gaps in the progression of PCOS pathogenesis.

## Introduction

Polycystic ovary syndrome (PCOS) is the most common endocrine disorder among women of reproductive age, with an estimated global prevalence of 4–20% depending on population and diagnostic criteria (Deswal *et al.* 2020). Despite this, PCOS remains challenging to treat due to its multifactorial etiology and heterogeneous presentation. Current diagnoses typically employ the Rotterdam criteria, which require at least two of three key symptoms: i) irregular menstrual cycle and ovulatory dysfunction, ii) biochemical or clinical hyperandrogenism, and iii) polycystic ovarian morphology (Rotterdam ESHRE/ASRM-Sponsored PCOS Consensus Workshop Group 2004, Teede *et al.* 2018). Based on these criteria, patients can be classified as one of four phenotypes (types A–D), which present varying endocrine, reproductive and metabolic symptoms (Table 1). PCOS is associated with numerous comorbidities, including infertility, pregnancy complications, psychological disorders such as anxiety and depression, and metabolic dysfunction that may include insulin resistance, elevated insulin levels (i.e., hyperinsulinemia), obesity, dyslipidemia and increased risks of type 2 diabetes and cardiovascular disease (Dumesic *et al.* 2015).

**Table 1:** Summary of symptoms associated with PCOS phenotypes A–D.

PCOS phenotype	Diagnostic features			Likely presence of hyperinsulinemia (Ezeh *et al.* 2022)	Estimated prevalence of insulin resistance (Moghetti *et al.* 2013)
	Ovulatory dysfunction	Hyperandrogenism	Polycystic ovarian morphology		
A	✓	✓	✓	✓	80%
B	✓	✓		✓	80%
C		✓	✓	✓	65%
D	✓		✓		38%

Insulin resistance and hyperinsulinemia – present in ∼60–95% (Tosi *et al.* 2017) of patients with PCOS – play a fundamental role in its pathogenesis by perpetuating hyperandrogenism and ovarian dysfunction. Elevated insulin levels are typically described as a compensatory β-cell response to insulin resistance in peripheral tissues (Reaven 1988, 2005), yet it is difficult to delineate the temporal relationship between insulin resistance and hyperinsulinemia, as these conditions are closely interlinked and often detected together. Importantly, hyperinsulinemia can be an upstream driver of insulin resistance in metabolic syndrome and type 2 diabetes (Shanik *et al.* 2008, Tricò *et al.* 2018, Nolan & Prentki 2019, Johnson 2021). Therefore, we wished to examine each of the alternative cause-and-effect frameworks for insulin resistance and hyperinsulinemia in PCOS.

## Insulin signaling

Insulin is a metabolic hormone best known for regulating glucose homeostasis, although it also affects lipid flux, protein synthesis, autophagy, cell growth and proliferation, stress responses, ovarian steroidogenesis and folliculogenesis (Saltiel & Kahn 2001, Athar *et al.* 2024). Insulin is secreted from pancreatic β-cells in response to nutrient stimuli such as elevated glucose, amino acids and fatty acids. Insulin secretion is also regulated by many other hormones, paracrine and autocrine factors, and neurotransmitters (Huising 2020, Henquin 2021)

**Figure 1:**
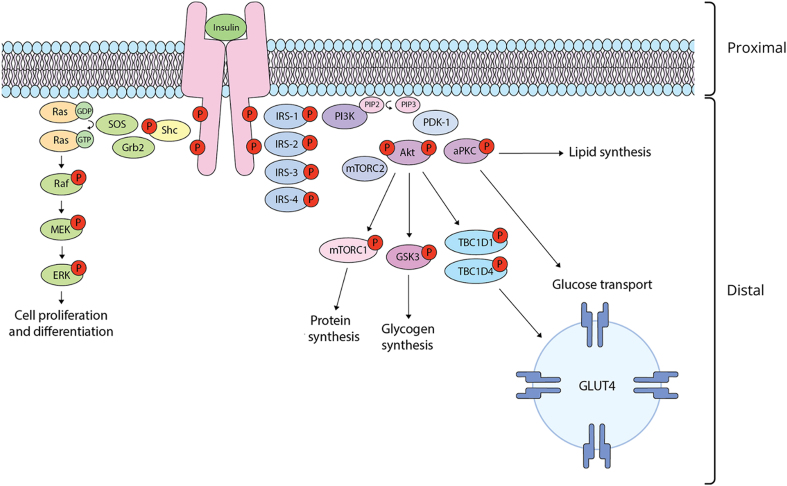
Simplified insulin signal transduction cascades. Binding of insulin to the tyrosine kinase receptor (i.e., proximal insulin signaling) induces dimerization and autophosphorylation of the receptor beta subunit, creating docking sites for adapter proteins that initiate the mitogenic MAPK/ERK cascade and/or the metabolic PI3K/Akt cascade. Phosphorylation of Shc by the insulin receptor allows for the binding of Grb2, an adapter protein that complexes with son of sevenless (SOS), a guanylyl exchange factor. SOS promotes GDP/GTP exchange on Ras, a GTPase that activates a serine/threonine kinase cascade, which results in phosphorylation of ERK. Once phosphorylated, ERK can enter the nucleus and activate various transcription factors and mitogen-activated protein kinases, leading to coordination of cell proliferation and differentiation. The PI3K/Akt cascade is initiated by the recruitment and activation of PI3K by IRSs IRS1/2. PI3K phosphorylates phosphatidylinositol 4,5-bisphosphate (PIP_2_), producing phosphatidylinositol 3,4,5-triphosphate (PIP_3_). Accumulation of PIP_3_ creates docking sites for phosphoinositide-dependent kinase 1 (PDK1), which phosphorylates and activates Akt. Akt performs many downstream functions. For instance, Akt activates protein synthesis through phosphorylation of mTORC1/2, glycogen synthesis through phosphorylation of GSK3, lipid synthesis through activation of aPKC and glucose transport by phosphorylating TBC1D1/4, which promote translocation of GLUT4-containing vesicles to the cell membrane, ultimately leading to glucose uptake into the cell. A full color version of this figure is available at https://doi.org/10.1530/JOE-24-0269.

Although there is some functional overlap, insulin actions can be generally divided between two main branches of insulin signaling: a mitogenic cascade, and a metabolic cascade that regulates glucose transport and macromolecule synthesis (Fig. 1; Cheatham & Kahn 1995). Insulin initiates these cascades by causing dimerization and autophosphorylation of its tyrosine kinase receptor. This is followed by phosphorylation of signaling intermediaries, such as Shc and insulin receptor substrate (IRS) proteins. Phosphorylation of Shc establishes mitogenic actions through recruiting Ras, a GTPase. Activated Ras triggers the phosphorylation and activation of mitogen-activated protein kinase (MAPK), which initiates the MAPK/ERK cascade that promotes cell proliferation and differentiation. The metabolic actions of insulin are largely modulated by the phosphatidylinositol 3-kinase (PI3K) and Akt cascade. Phosphorylation of IRS-1 and/or IRS-2 leads to recruitment and activation of PI3K, which catalyzes the synthesis of phosphatidylinositol-3,4,5-triphosphate (PIP_3_). PIP_3_ accumulation creates docking sites for Akt and phosphoinositide-dependent kinase 1 (PDK1), which phosphorylates and activates Akt, a serine/threonine kinase that mediates many metabolic actions of insulin (Saltiel & Kahn 2001).

Among its other effects, insulin is a major player in regulating glucose homeostasis. In skeletal muscle, Akt is involved in inducing translocation of GLUT4 glucose transporters to the cell membrane for glucose uptake. Akt also promotes glycolysis, glucose utilization, and glycogen synthesis and storage in myocytes (Saltiel & Kahn 2001). In adipocytes, Akt mediates glucose uptake via GLUT4 translocation, in addition to inhibiting lipolysis through repressing lipolytic enzymes and upregulating lipogenic enzymes (Saltiel & Kahn 2001). In the liver, glucose transport is not dependent on insulin, but hepatocyte insulin signaling increases synthesis of glycogen, lipids and proteins while inhibiting glucose production (Saltiel & Kahn 2001). Insulin-stimulated responses in other tissues, such as the suppression of free fatty acid release by adipocytes, may also indirectly reduce hepatic glucose production (Bergman & Iyer 2017). Through these collective effects in its canonical target tissues, insulin reduces postprandial glucose levels (Saltiel & Kahn 2001). However, insulin is not the sole regulator of glucose levels. For instance, direct actions of glucagon, cortisol and catecholamines in the liver mobilize glucose by promoting hepatic glycogenolysis and/or gluconeogenesis, among their other effects. Therefore, insulin-independent mechanisms also contribute to the fine-tuned controls over glucose homeostasis, in which central and peripheral regulatory points are integrated to maintain blood glucose within a narrow range (Lam *et al.* 2009, Nirmalan & Nirmalan 2023).

## Defining insulin resistance and hyperinsulinemia

With insulin resistance, defectiveness or dysregulation of an insulin signaling component leads to impaired glucose metabolism. Clinically, insulin resistance is defined by a requirement for excessive insulin to mediate the appropriate response to a glucose load, or higher-than-expected blood glucose levels relative to insulin. Hyperinsulinemic-euglycemic clamps provide the most direct measure of insulin resistance, although they are labor intensive and challenging to implement in clinical practice. This method consists of infusing insulin above the normal fasting level while administering a dextrose solution to maintain blood glucose within the euglycemic range, and measuring whole-body glucose uptake. However, the supraphysiological insulin levels of the clamp may not accurately reflect physiological glucose and insulin dynamics (Muniyappa *et al.* 2008). The minimal model analysis of Frequently Sampled Intravenous Glucose Tolerance Test (FSIVGTT) is often used in both clinical and research settings. FSIVGTT involves administering an intravenous bolus of glucose followed by injection of insulin after 20 min, with blood samples drawn over 180 min; the insulin sensitivity index (*S_I_*) calculated with the minimal model analysis provides an estimate of whole-body insulin sensitivity (Bergman *et al.* 1987, Bergman 2021). The homeostasis model assessment of insulin resistance (HOMA-IR) and quantitative insulin sensitivity check index (QUICKI) are both surrogate indices of insulin resistance that are mathematically derived from fasting glucose and insulin levels. These models are minimally invasive, but they are not the most accurate measurement of insulin resistance, and they may not distinguish insulin resistance from hyperinsulinemia itself. Indices of insulin sensitivity can also be calculated from insulin and glucose dynamics during an oral glucose tolerance test (Muniyappa *et al.* 2008).

At a molecular level, insulin resistance is defined as an impaired cellular response to insulin, resulting in a decreased capacity for glucose disposal in response to the insulin signal. This is often due to defects that are distal in the insulin signaling cascade, such as at the level of effector proteins or GLUT4 translocation (James *et al.* 2021). Tissue insulin sensitivity is assessed by quantifying phosphorylation of signaling proteins (such as Akt, PI3K, IRS-1 and INSR) after insulin administration. Depending on where the molecular defect(s) lie within the signaling pathway, insulin resistance could affect many metabolic and mitotic processes, or might instead have restricted effects. Clinical definitions of insulin resistance primarily concern the impacts of insulin on glucose homeostasis, while disregarding other insulin-mediated cellular processes that may or may not be functioning normally. However, the breadth of insulin resistance is highly variable, and there may only be altered glucose metabolism within certain tissues rather than generalized impairment of all insulin signaling. Moreover, any tissue or biological process that remains insulin-responsive in this state may be exposed to a persistent elevation in insulin stimulus.

There is a range of basal insulin levels within a population, and hyperinsulinemia, or elevated circulating insulin without hypoglycemia, is often detected when there is clinically defined insulin resistance. Notably, hyperinsulinemia can also be observed in the absence of hyperinsulinemic-euglycemic clamp-measured insulin resistance, and it may be more prevalent than insulin resistance among nondiabetic obese individuals (Ferrannini *et al.* 1997, Tricò *et al.* 2018). Although insulin lowers postprandial blood glucose, the chronic hyperinsulinemia that can be detected in metabolic disorders such as obesity and PCOS does not induce hypoglycemia (Thomas *et al.* 2019). This differs from rarer situations of pronouncedly excessive insulin secretion, as with insulinomas (i.e., insulin-producing pancreatic tumors), which are the most common endogenous causes of hypoglycemia (largely due to decreased hepatic glucose output rather than increased insulin-stimulated glucose utilization; Grant 2005). Therefore, hyperinsulinemia is often clinically defined by elevated fasting insulin relative to average levels for a particular age and health status, without hypoglycemia (Thomas *et al.* 2019).

Clinical tests may conflate hyperinsulinemia and insulin resistance, and their causal relationship is often unresolved. A tight link between insulin sensitivity and insulin secretion is thought to minimize changes in glucose tolerance; a shift in insulin sensitivity is tied to a responsive change in glucose-stimulated insulin release, and vice versa (Bergman 2024). As a result, it is challenging to temporally delineate the sequence of these metabolic changes. The dominant paradigm posits that hyperglycemia caused by insulin resistance amplifies β-cell secretion of insulin, resulting in compensatory hyperinsulinemia. Conversely, an alternate framework describes hyperinsulinemia as a primary defect and insulin resistance as a protective response of tissues against insulin-induced nutrient overload and metabolic stress (Nolan & Prentki 2019). Together with the imprecise clinical definitions of hyperinsulinemia and insulin resistance, these two contrasting perspectives highlight the complexities in defining causality between insulin resistance and hyperinsulinemia.

**Figure 2:**
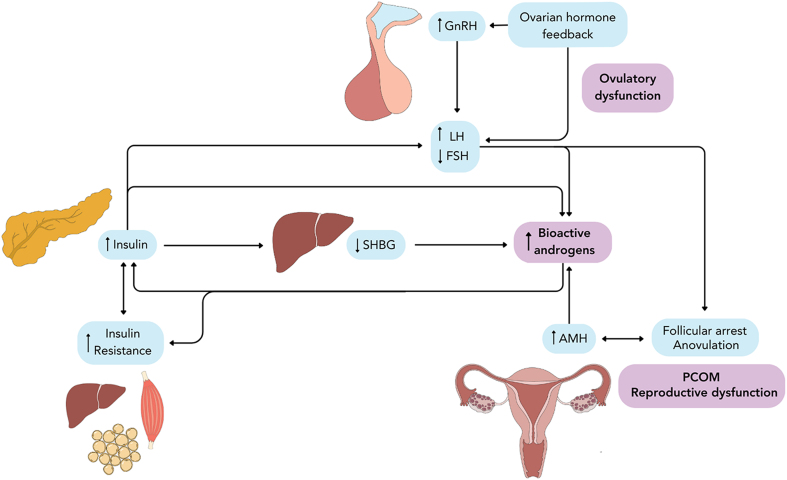
Pathophysiology of PCOS. PCOS is best defined by the presence of ovulatory dysfunction (e.g., oligo/anovulation), hyperandrogenemia, and/or polycystic ovarian morphology (PCOM), symptoms which are induced by alterations of the hypothalamic–pituitary–ovary axis and metabolic dysfunctions. Hyperactive GnRH-releasing neurons lead to hypersecretion of LH and reduced FSH secretion by the anterior pituitary gland. LH signals to ovarian theca cells to increase androgen biosynthesis, while granulosa cell aromatization of androgens is diminished due to relative FSH deficiency, resulting in hyperandrogenemia. Elevated insulin levels due to causes such as compensatory β-cell responses to insulin resistance work synergistically with LH to promote ovarian steroidogenesis and suppress hepatic production of SHBG, thus exacerbating hyperandrogenemia by increasing levels of bioavailable androgens. Simultaneously, androgens can worsen insulin resistance and increase insulin production, creating a self-perpetuating cycle of hyperinsulinemia and hyperandrogenemia. Elevated androgen levels, combined with excessive AMH production by immature follicles, contribute toward arresting follicle development, thus leading to polycystic ovarian morphology and anovulation. A full color version of this figure is available at https://doi.org/10.1530/JOE-24-0269.

In PCOS, insulin resistance is often perceived as the upstream metabolic disturbance that induces an elevation in insulin levels, and secondarily raised insulin augments testosterone production and bioavailability among other reproductive and metabolic effects (Fig. 2; Teede *et al.* 2010). However, evolving perspectives in other fields and data from PCOS patients and animal models point to a need for this framework to be examined more closely. Here, we aimed to assess the relationship between insulin resistance and hyperinsulinemia in the context of PCOS, examining both the theory of hyperinsulinemia as a compensatory response to insulin resistance and the alternative concept of hyperinsulinemia as a root cause that leads to insulin resistance and other repercussions.

## PCOS pathogenesis: roles of androgens and insulin

A constellation of changed reproductive hormone levels underlies PCOS pathogenesis (Fig. 2). Gonadotropin-releasing hormone (GnRH) neurons are hyperactive and have increased pulsatile frequency in PCOS, resulting in elevated luteinizing hormone (LH) and reduced follicle-stimulating hormone (FSH) secretion by the pituitary gland (Waldstreicher *et al.* 1988, Silva *et al.* 2023). Coupled with intrinsic theca cell defects, the elevation in LH stimulates greater theca cell production of androgens, such as androstenedione and testosterone, while relative FSH deficiency reduces the granulosa cell-mediated aromatization of androgens to estradiol (Gilling-Smith *et al.* 1994, 1997). In addition, abnormal responses to adrenocorticotropic hormone can lead to hypersecretion of androgens by the adrenal glands (McGee *et al.* 2012, Maas *et al.* 2016). Hyperandrogenism in PCOS is compounded by elevated anti-Müllerian hormone (AMH) levels. AMH inhibits FSH-stimulated granulosa cell aromatase activity (Grossman *et al.* 2008), and the resulting hyperandrogenemia increases expression of FSH receptors, leading to follicle recruitment and pre-antral follicle growth (Luo & Wiltbank 2006, Catteau-Jonard *et al.* 2008). Follicle development is arrested in response to the combination of high androgens and the excessive AMH secreted by many immature follicles; increased numbers of growing pre-antral follicles cause polycystic ovarian morphology, and these folliculogenesis defects are also associated with impaired dominant follicle selection and anovulation (Catteau-Jonard *et al.* 2008, Franks *et al.* 2008, Alebić *et al.* 2015, Chen *et al.* 2015). Altered levels of AMH and other ovary-produced hormones further perpetuate GnRH-dependent LH pulsatility and secretion (Blank *et al.* 2006, Cimino *et al.* 2016). For instance, elevated androgens reduce the sensitivity of the hypothalamus to negative feedback inhibition by the ovarian steroids estradiol and progesterone, thus contributing to the increased LH pulse frequency (Eagleson *et al.* 2000). Indeed, central androgen signaling in neurons and/or pituitary may play an integral role in causing PCOS, as evidenced by the protection against central and ovarian PCOS-like phenotypes afforded to mice with androgen receptors (AR) knocked out of these tissues in dihydrotestosterone (DHT)- or letrozole-induced PCOS disease models (Caldwell *et al.* 2017, Wang *et al.* 2019, Decourt *et al.* 2023, Ubba *et al.* 2024).

Elevated insulin exacerbates many aspects of PCOS pathophysiology. At the level of the central nervous system, insulin receptors are widely expressed in the brain, and insulin likely plays a role in regulating the hypothalamic–pituitary–ovarian axis through such means as stimulating GnRH expression (Athar *et al.* 2024). Exogenous insulin administration acutely increases circulating LH levels in female mice, while *INSR* knockout in either GnRH neurons or the pituitary protects against obesity-associated infertility and lowers basal LH levels (Brothers *et al.* 2010, DiVall *et al.* 2015). While this indicates that insulin excess can act centrally to worsen reproductive function, specific effects may not be consistent in humans. For instance, an acute, supraphysiological infusion of insulin under a hyperinsulinemic-euglycemic clamp does not appear to elevate LH in obese PCOS patients or non-PCOS controls; instead, it sometimes has a suppressive effect (Dunaif & Graf 1989, Patel *et al.* 2003, Lawson *et al.* 2008). Interestingly, while PCOS imparts high LH levels and pulse amplitudes, obesity is associated with a reduction in LH levels and pulse amplitudes in humans, including in women with PCOS (Eng *et al.* 2024).

Insulin acts as a co-gonadotroph in the ovary, where activation of the PI3K/AKT signaling cascade is also involved in regulating ovarian follicle survival, growth and activation (Athar *et al.* 2024). Insulin works synergistically with LH to increase theca cell production of androgens and upregulate theca cell LH receptors, effects which are pronounced in women with PCOS (Nestler *et al.* 1998, Tosi *et al.* 2012). Insulin also augments FSH-stimulated granulosa cell production of estradiol and progesterone (Willis *et al.* 1996), although FSH stimulation is relatively diminished with PCOS. In addition to promoting steroidogenesis in the ovary, insulin appears to play a role in potentiating adrenal androgen synthesis, which is significantly upregulated under a hyperinsulinemic-euglycemic clamp in hyperandrogenic women (Tosi *et al.* 2011). Furthermore, insulin decreases hepatic production of sex hormone-binding globulin (SHBG) in women with PCOS, thus leading to increased circulating levels of bioactive testosterone (Baillargeon & Carpentier 2007). Despite these actions, chronic hyperinsulinemia alone may not elevate androgen levels in the absence of other predisposing defects. Experimentally inducing hyperinsulinemia via an extended period of exogenous insulin administration did not stimulate hyperandrogenemia in adult female non-human primates (Zhou *et al.* 2022) or adult female rats (Poretsky *et al.* 1988), although interpretation is complicated by the caveat that experimentally induced hyperinsulinemia can cause compensatory insulin receptor downregulation in relevant tissues, such as the ovaries (Poretsky *et al.* 1988). Adding to the complexity, testosterone can itself induce insulin hypersecretion (Mishra *et al.* 2018, Navarro *et al.* 2018, Xu *et al.* 2019). The bilateral relationship between hyperinsulinemia and hyperandrogenemia is a key mechanism of PCOS pathogenesis, but the origins and temporal relationship between these features remain to be defined (Moghetti & Tosi 2021).

## Insulin resistance in PCOS

### Insulin resistance causes compensatory hyperinsulinemia outside of PCOS conditions

In metabolic conditions such as type 2 diabetes, obesity is often described as a cause of insulin resistance. Increased adipose tissue mass and adipocyte dysfunction promote greater release of inflammatory cytokines, free fatty acids and damaging adipokines, and increase ectopic lipid deposition in the liver, muscle and pancreas (James *et al.* 2021). Collectively, these changes induce insulin resistance in the liver and muscle, which leads to elevated blood glucose and subsequent insulin overproduction.

Genetic mutations in components of the insulin signaling pathway can also contribute to insulin resistance and induce secondary hyperinsulinemia. Notably, several mutations within insulin signaling molecules have been uncovered in patients with elevated insulin levels, providing plausible evidence that insulin resistance can precede hyperinsulinemia (Parker *et al.* 2011, Kushi *et al.* 2021). For instance, a missense mutation in the gene encoding AKT2/PKB resulted in hyperinsulinemia and type 2 diabetes (George *et al.* 2004). Similarly, familial studies of patients with acanthosis nigricans and hyperinsulinemia revealed a loss-of-function mutation in *TBC1D4*, which encodes for a protein involved in GLUT4 trafficking; loss of its function causes severe insulin resistance (Dash *et al.* 2009). Mice with adipocyte-selective GLUT4 reduction showed insulin resistance in hyperinsulinemic-euglycemic clamps, impaired glucose tolerance and hyperinsulinemia (Abel *et al.* 2001). This mouse model also exhibited reduced PI3K activity in myocytes and hepatocytes, suggesting that adipose tissue insulin resistance may induce or exacerbate insulin resistance in other tissues (Abel *et al.* 2001). Similarly, liver-specific insulin receptor knockout causes severe insulin resistance, and it also induces elevated insulin levels that arise from increased pancreatic insulin content and reduced insulin clearance (Michael *et al.* 2000). Together, these examples indicate that insulin resistance caused by a molecular defect in insulin signaling can lead to secondary hyperinsulinemia. However, these specific genetic anomalies may not be widely representative of the form of insulin resistance in obesity, type 2 diabetes or PCOS.

### Potential causes of insulin resistance in PCOS

The high prevalence of insulin resistance in PCOS patients was first described by Dunaif *et al.* (1989), and it is now recognized as a cardinal feature of the disorder. Insulin resistance in hyperinsulinemic-euglycemic clamps is most common in PCOS types A and B, intermediate in type C and least common in type D (Table 1; Moghetti *et al.* 2013). This can present as impairments in the metabolic and/or mitotic branches of insulin signaling in various tissues, including canonical insulin targets (Diamanti-Kandarakis & Dunaif 2012) and nonclassical targets such as ovaries (Rice *et al.* 2005, Guo *et al.* 2022). For many years, insulin resistance was characterized as a consequence of obesity in PCOS patients, and not as a primary defect (Rachoń & Teede 2010). However, insulin resistance has been observed in both normal-weight and obese patients, which contradicts the theory of obesity as the sole driver of insulin resistance (Stepto *et al.* 2013).

Across the past three decades, an assortment of molecular defects within the insulin signaling pathway have been identified in PCOS patients. Genome-wide association studies and candidate gene association studies provided some evidence for single nucleotide polymorphisms of the insulin receptor (*INSR*) gene that may be associated with PCOS, although the associations were not found across all studies, and functional impacts of the genetic changes are unclear (McAllister *et al.* 2015). Similarly, altered insulin receptor phosphorylation patterns were identified in skin fibroblasts and skeletal muscle of a subset of PCOS patients. Approximately 50% of PCOS subjects exhibited decreased insulin-induced tyrosine phosphorylation of the insulin receptor beta subunit, pointing to a post-binding defect (Dunaif *et al.* 1995). Nonetheless, a definitive causal point of insulin resistance has not been identified in PCOS patients, due in part to the inconsistencies in specific aberrations that have been detected across different studies, patients and tissues.

The molecular mechanisms that cause insulin resistance in PCOS likely vary depending on PCOS phenotype and etiology. It is also challenging to differentiate PCOS-specific changes from the obesity-induced alterations in insulin sensitivity for the ∼61% (Lim *et al.* 2012) of PCOS patients who are also overweight or obese. It is probable that a combination of factors contribute to insulin resistance with PCOS, including genetic and epigenetic programming, crosstalk from other metabolic pathways and tissues, hyperandrogenemia, mitochondrial dysfunction, altered adipokine secretion, ectopic lipid accumulation and other features of metabolic stress (Stepto *et al.* 2019, Bril *et al.* 2024).

#### Adipose tissue and insulin resistance in PCOS

Adipose tissue plays a key role in insulin resistance that extends beyond its contributions to glucose disposal (Bril *et al.* 2024). Compared to BMI-matched controls, PCOS patients (even those who may have normal weights based on BMI) typically have increased intra-abdominal fat mass and whole-body fat-to-lean ratio, which is positively correlated with whole-body insulin resistance, fasting insulin levels and circulating androgens (Ezeh *et al.* 2014, Dumesic *et al.* 2016). In addition, PCOS patients ranging from normal-weight to obese show evidence of impaired adipocyte functioning compared to BMI-matched controls, including increased proportions of enlarged and/or small subcutaneous abdominal adipocytes and altered adipokine profiles (Mannerås-Holm *et al.* 2011, McLaughlin *et al.* 2014, Dumesic *et al.* 2016, Lin *et al.* 2021). Increased intra-abdominal fat mass and adipocyte dysfunction can lead to elevated release of free fatty acids and ectopic lipid deposition, and a shifted secretory profile toward increased secretion of pro-inflammatory cytokines and adipokines, which reduce insulin sensitivity by suppressing insulin signal transduction (Shulman 2014, Ahmed *et al.* 2021, Lin *et al.* 2021). Meanwhile, levels of adiponectin, an insulin-sensitizing adipokine, are significantly reduced in lean PCOS patients (Hansen *et al.* 2019). In a DHT-induced mouse model of PCOS, overexpression of adiponectin attenuated insulin resistance and glucose intolerance, while mice with adiponectin knockout showed decreased insulin sensitivity (Benrick *et al.* 2017). This suggests a role for low adiponectin in the development of PCOS-related metabolic disturbances.

Distal insulin signaling perturbations may also contribute to insulin resistance in adipose tissue. Adipocytes of PCOS patients were found to be comparable to controls for insulin receptor binding and kinase activity (Ciaraldi *et al.* 1992), and for total protein and phosphorylation levels of IRS-1, PI3K and Akt (Chen *et al.* 2013). However, a pronounced reduction in GLUT4 content in adipocytes of both normal-weight and obese PCOS patients reflects a decreased capacity for insulin-stimulated glucose uptake in this tissue (Rosenbaum *et al.* 1993). Adipocyte *GLUT4* mRNA levels are negatively associated with HOMA-IR-determined insulin resistance, and these low *GLUT4* levels are not compensated by upregulated *GLUT1* expression (Ezeh *et al.* 2019). The reduction of GLUT4 in PCOS adipocytes may be due in part to an upregulation of miRNA-93, which represses *GLUT4* transcription (Chen *et al.* 2013). In adipocytes of some PCOS patients, insulin-mediated phosphorylation patterns of glycogen synthase kinase (GSK)-3 are altered, resulting in hyperactivation. GSK3 inhibits glycogen synthase, thus its constitutive activation would prevent normal glycogen synthesis (Chang *et al.* 2008).

Adipose tissue insulin resistance in PCOS is positively correlated with both serum androgen levels and whole-body insulin resistance (Dumesic *et al.* 2019). Testosterone can impair insulin-stimulated phosphorylation of an atypical protein kinase C (aPKCζ) in human adipocytes, thereby reducing glucose uptake (Corbould 2007). This may not extend to mitotic insulin signaling effects, as extracellular signal-regulated kinase (ERK) phosphorylation was unchanged in adipocytes cultured with testosterone (Corbould 2007). On top of increased circulating testosterone, PCOS is also associated with more intra-adipose androgens. Subcutaneous adipose tissue from obese women with PCOS has increased expression of the androgen-activating enzyme aldo-ketoreductase type 1 C3 (AKR1C3), together with elevated intra-adipocyte androgen levels (O’Reilly *et al.* 2017). This local elevation in androgens appears capable of suppressing lipolysis and promoting lipid accumulation, thus worsening insulin resistance (O’Reilly *et al.* 2017). Interestingly, exposing primary female subcutaneous adipocytes to insulin *in vitro* significantly increases *AKR1C3* mRNA expression, which points to the potential for elevated insulin to potentiate this rise in intra-adipose androgens and subsequent insulin resistance (O’Reilly *et al.* 2015).

#### Hepatic insulin resistance in PCOS

The liver is the main site of glycogen storage and insulin clearance, and is a key regulator of insulin resistance. Increased intra-abdominal adiposity and subsequent release of free fatty acids can lead to hepatic steatosis, or ectopic lipid accumulation in the liver, which often precedes the onset of insulin resistance in the liver, muscle and adipose tissue. This may be in part due to endocrine and paracrine effects of hepatokines such as fetuin A, SHBG and retinol-binding protein (Meex & Watt 2017). In PCOS, many of these hepatokines are dysregulated, although the physiological effects have yet to be determined (Stefanaki *et al.* 2023). Hepatic steatosis can also contribute to hepatic insulin resistance due to increased fatty acid flux. This elevates intracellular diacylglycerol, which eventually leads to impaired insulin signaling and dysregulated glycogen synthesis and gluconeogenesis in the liver (Shulman 2014).

High androgen levels contribute to obesity-associated hepatic steatosis and insulin resistance in PCOS models. Long-term DHT exposure in mice resulted in obesity, hepatic lipid accumulation, upregulation of inflammatory markers in the liver and mitochondrial dysregulation. Concurrently, levels of insulin receptor β subunit, IRS1 and Akt substrate of 160 kDa (AS160) were all decreased, pointing to hepatic insulin resistance (Cui *et al.* 2021). Simultaneous knockout of AR in both brain and adipose tissue ameliorated several metabolic features in a DHT-induced mouse model of PCOS, including increased adiposity, adipocyte hypertrophy and hepatic steatosis (Cox *et al.* 2020). Similarly, DHT-exposed mice with liver-specific AR knockout were protected against metabolic dysfunction compared to wild-type DHT-treated controls. Their livers maintained insulin sensitivity and had elevated Akt phosphorylation and PI3K activity compared to controls, which suggests a causative role of autonomous androgen signaling in the development of PCOS-related hepatic insulin resistance (Andrisse *et al.* 2021).

#### Skeletal muscle insulin resistance in PCOS

Skeletal muscle is the principal site of insulin-induced glucose uptake. Dunaif *et al.* (2001) detected reduced IRS-1-associated PI3K activity in skeletal muscle from obese PCOS patients, which could contribute toward the observed decrease in whole-body glucose disposal during a hyperinsulinemic-euglycemic clamp. The same molecular defects were not evident in skeletal muscle myotubes in culture, which suggests that the surrounding hormonal and metabolic milieu of PCOS helps drive skeletal muscle insulin resistance, although intrinsic defects could increase susceptibility (Corbould *et al.* 2005). Højlund *et al.* (2008) did not find a significant change in IRS-1-associated PI3K activity, but instead showed decreased insulin-stimulated phosphorylation of Akt at Ser^473^ and Thr^308^ in skeletal muscle of obese PCOS patients compared to BMI-matched controls, accompanied by insulin resistance in hyperinsulinemic-euglycemic clamps. The mitogenic actions of insulin signaling may also be impaired in the skeletal muscle of PCOS patients. Rajkhowa *et al.* (2009) found that while acute insulin stimulation of skeletal muscle from overweight PCOS patients was not associated with IRS-1 or Akt differences, activation of ERK1/2 was significantly reduced.

Molecular causes of skeletal muscle insulin resistance can be more elusive in lean individuals with PCOS. A recent examination of lean PCOS patients revealed no change in insulin-stimulated Akt phosphorylation in skeletal muscle, nor any difference in GLUT4 protein levels, despite whole-body insulin resistance in hyperinsulinemic-euglycemic clamps. Reduced insulin sensitivity was instead attributed to decreased AMPK phosphorylation, potentially due to lower adiponectin (Hansen *et al.* 2019). Furthermore, these PCOS patients had increased intramuscular lipid content, even though they were lean (Hansen *et al.* 2019). In a low-dose DHT mouse model of nonobese PCOS, skeletal muscle actually showed elevations in insulin-stimulated glucose transport, AKT phosphorylation and GLUT4 protein levels, unlike the molecular insulin resistance observed in the livers and white adipose tissue of these mice (Andrisse *et al.* 2018).

Nonetheless, hyperandrogenemia may make some contributions to skeletal muscle insulin resistance in PCOS. For instance, when rat skeletal muscle myotubes are exposed to testosterone for 16 h, they exhibit increased insulin-induced serine phosphorylation of IRS-1, which is thought to uncouple IRS-1 signal transduction to PI3K, and thus result in insulin resistance (Allemand *et al.* 2009). In addition, DHT exposure in mice can lower the proportion of insulin-sensitive type I muscle fibers, mediated by skeletal muscle AR (Stener-Victorin *et al.* 2024). This is consistent with the shifted muscle fiber type profile that was revealed in a global proteomic analysis of hyperandrogenic PCOS patients (Stener-Victorin *et al.* 2024). Therefore, while an intrinsic molecular aberration has not been consistently associated with skeletal muscle insulin resistance in PCOS, it seems that physiological features of PCOS are able to instigate some reductions in insulin sensitivity in this tissue.

#### Insulin responsiveness in the PCOS hypothalamus–pituitary–ovarian axis

Insulin can act centrally to regulate hormones of the hypothalamic–pituitary–ovarian axis, although it remains to be determined whether there is altered responsiveness of these effects to insulin with PCOS. In normal conditions, insulin stimulates GnRH gene expression in mouse GnRH neurons of the hypothalamus (Kim *et al.* 2005). It can also act directly on the anterior pituitary to increase LH secretion in rodents (Adashi *et al.* 1981, Xia *et al.* 2001), although acutely infusing high insulin does not appear to further elevate LH in obese women with PCOS (Dunaif & Graf 1989, Patel *et al.* 2003, Lawson *et al.* 2008). It is unclear whether there are changes in hypothalamic or pituitary insulin sensitivity that impact the reproductive axis in the presence of peripheral insulin resistance and/or PCOS (Sliwowska *et al.* 2014, Ozgen Saydam & Yildiz 2021). However, low-dose DHT exposure causes an increase in hypothalamic AKT phosphorylation despite raising peripheral glucose levels (Ubba *et al.* 2023), and preservation of AKT phosphorylation levels in pituitary tissue similarly contrasts with the hepatic and adipose tissue insulin resistance of low-dose DHT-treated mice (Andrisse *et al.* 2018). These molecular observations suggest a maintenance of central insulin sensitivity despite peripheral insulin resistance in this PCOS mouse model. Interestingly, neuronal androgen signaling might contribute toward driving peripheral insulin resistance in PCOS, since kisspeptin neuron-specific AR knockout improves whole-body insulin-stimulated glucose disposal (and other PCOS-like traits) in a letrozole-induced mouse model of PCOS (Decourt *et al.* 2023).

At the level of the ovaries, androgen production is a cardinal feature of PCOS pathogenesis that remains highly responsive to insulin. Insulin can act directly on ovarian theca cells to augment gene expression and activity of 17-alpha hydroxylase/CYP17, a key enzyme for androgen production (Munir *et al.* 2004). These effects are regulated by PI3K activation (Munir *et al.* 2004), and theca cell-specific deletion of the insulin receptor is sufficient to prevent a rise in ovarian *CYP17* levels and androgen production in high-fat diet-fed mice (Wu *et al.* 2014), which highlights the direct thecal cell role for insulin in promoting androgen synthesis. Notably, PCOS theca cells show more insulin-stimulated testosterone biosynthesis than non-PCOS theca cells, demonstrating that theca cells have increased sensitivity to the steroidogenic effects of insulin in women with PCOS (Nestler *et al.* 1998). Indeed, Yen *et al.* (2004) observed higher levels of IRS-1 and IRS-2 in theca cells from overweight PCOS subjects compared to non-PCOS controls. On the other hand, cultured ovarian granulosa cells of PCOS patients demonstrated decreased GLUT4 expression and reduced insulin-stimulated glucose uptake (Guo *et al.* 2022). However, this form of insulin resistance seems limited to the metabolic actions of insulin in PCOS granulosa cells, as they maintain a normal level of insulin-stimulated progesterone production, even when there is reduced insulin-regulated glucose metabolism (Willis *et al.* 1996, Rice *et al.* 2005). Overall, PCOS ovaries show continued or elevated sensitivity to the steroidogenic effects of insulin. This contributes to PCOS pathogenesis, as increased ovarian androgen production due to hyperinsulinemia augments hyperandrogenism and the resulting reproductive and metabolic traits of PCOS.

Altogether, it seems that a subset of insulin signaling effects – including those related to androgen production – tend to remain insulin-responsive with PCOS, despite the potential for reduced insulin sensitivity with respect to some metabolic processes. Both lean and obese individuals with PCOS may have reduced insulin-stimulated glucose disposal. This has been linked to insulin signaling aberrations in adipose tissue, liver and/or skeletal muscle, representing an assortment of molecular changes that may be acquired, although this does not preclude the possibility of intrinsic defects. Other PCOS features such as increased intra-abdominal adiposity, lower adiponectin and hyperandrogenemia are probable contributors to impaired insulin responsiveness in these metabolic tissues. Whole-body insulin resistance is rarely detected without concomitant hyperinsulinemia in PCOS, which makes it difficult to establish the temporal and causal relationship between these two features. However, it seems that insulin resistance can be caused by a range of PCOS sequelae; in some cases, a key upstream contributor may be hyperinsulinemia itself.

## Hyperinsulinemia in PCOS

### Hyperinsulinemia can cause or exacerbate insulin resistance outside of PCOS conditions

There is ongoing discourse surrounding a causative role for hyperinsulinemia in the development of insulin resistance in obesity and type 2 diabetes (Shanik *et al.* 2008, Nolan & Prentki 2019, Johnson 2021). Many lines of evidence from human subjects and animal models support this relationship. For instance, high-fat feeding in mice can induce β-cell proliferation after 3 days, long before insulin resistance is evident (Mosser *et al.* 2015). Mice transfected with extra copies of the human insulin gene to induce hyperinsulinemia became insulin resistant and showed evidence of insulin receptor downregulation in their livers (Marbán & Roth 1996). Conversely, mouse models with genetically reduced insulin have shown that preventing hyperinsulinemia mitigates age-associated insulin resistance and high-fat diet-induced obesity (Mehran *et al.* 2012, Templeman *et al.* 2015, Templeman *et al.* 2017). Similarly, lowering insulin via low-dose streptozotocin (an agent that ablates β-cells) or diazoxide (a drug that decreases insulin secretion by activating ATP-sensitive potassium channels) can lead to improved insulin sensitivity in obese mouse models (Gray *et al.* 2010, Pedersen *et al.* 2015).

In humans, examples of hyperinsulinemia preceding insulin resistance can be found in certain pathologies, such as insulinomas. Glucose clamp studies of patients with insulinomas demonstrated that insulin resistance can develop in response to elevated insulin (Pontiroli *et al.* 1990). Experimentally inducing hyperinsulinemia through insulin infusion also reduces whole-body glucose utilization in humans (Rizza *et al.* 1985). At a cellular level, it is widely accepted that hypersecretion of a molecule can lead to desensitization of its signaling pathway, primarily through downregulation of the receptor. This concept also applies to insulin, which can downregulate its responses by causing receptor degradation and reduced receptor mRNA expression (Okabayashi *et al.* 1989). Hyperinsulinemia can also downregulate signaling at the level of effector molecules, disrupting phosphorylation of IRS-1, PI3K, Akt and PKC (Lu *et al.* 2018). In particular, prolonged insulin exposure of human skeletal muscle cell cultures caused impaired insulin-stimulated glucose uptake and decreased Akt and IRS-1 phosphorylation (Turner *et al.* 2020). Collectively, this evidence points to a paradigm in which hyperinsulinemia may precede and can directly cause insulin resistance.

### Potential causes of hyperinsulinemia in PCOS

Although insulin resistance might lead to an increase in glucose-induced insulin secretion in some PCOS patients, there are also other causes of hyperinsulinemia. The molecular mechanisms that trigger hyperinsulinemia are not fully known and likely depend on genetic makeup and disease state. Hyperinsulinemia can develop due to β-cell proliferation or heightened responsiveness of β-cells to nutrient stimulation, potentially driven by chronic overnutrition (e.g., high-sugar, high-fat diets) triggering increased postprandial insulin secretion (Corkey 2012). On the other hand, elevated insulin in the fasted state can arise from inability to regulate basal secretion, resulting in sustained hyperinsulinemia without glucose stimulation. Fasting hyperinsulinemia may be caused by increased reactive oxygen species, which can rise in response to inflammation, or by excess long-chain acyl-CoA esters that similarly disrupt normal β-cell functioning (Corkey *et al.* 2021). Although fasted and fed hyperinsulinemia are typically discussed separately, they likely overlap mechanistically, and both contribute to metabolic disorders. For example, lean and obese PCOS patients exhibit higher fasting and 2 h insulin levels in an oral glucose tolerance test compared to lean controls, while exhibiting greater glucose-stimulated mononuclear cell (MNC)-derived nuclear factor κB activation and secretion of tumor necrosis factor-α (Malin *et al.* 2015). This points to a potential role of systemic inflammation in impairing β-cell function and promoting increased fasted and fed insulin secretion in PCOS.

Insulin secretion is also regulated by paracrine and autocrine factors, neurotransmitters, and other hormones, which can upregulate insulin secretion even in the absence of nutrient signals (Henquin 2021). Interestingly, glucagon, the main counterregulatory hormone to insulin, has paracrine actions on β-cells that increase insulin secretion during states of hyperglycemia (Gray *et al.* 2023). Obese PCOS patients and hyperinsulinemic PCOS patients demonstrate an exaggerated insulinotropic effect of glucagon, with significantly higher insulin levels following glucagon infusion compared to lean PCOS patients and normo-ovulatory controls (Ciampelli *et al.* 1998). During experimentally-induced hypoglycemia, obese PCOS patients exhibited three-fold greater glucagon secretion than BMI-matched controls (Sam *et al.* 2017). This indicates that glucagon secretion may be dysregulated in some PCOS patients, which could have the dual role of countering hypoglycemia while also contributing to hyperinsulinemia via the stimulatory effects of paracrine glucagon signaling on insulin secretion.

Hyperandrogenemia is also likely to promote β-cell insulin hypersecretion in PCOS. The positive correlative relationship between androgen (androstenedione and testosterone) and insulin levels is well-established, and all of these hormones are elevated in PCOS patients (Burghen *et al.* 1980). DHT-treated mice exhibit postprandial insulin hypersecretion and reduced insulin sensitivity compared to controls, followed by β-cell failure (Navarro *et al.* 2018). However, these abnormalities are not observed in DHT-treated mice with AR knockout in β-cells or hypothalamic neurons, indicating that androgens have both central and β-cell-autonomous effects on insulin secretion and signaling (Navarro *et al.* 2018). Following chronic DHT exposure, cultured female mouse and human β-cells exhibit insulin hypersecretion in response to glucose, followed by oxidative damage-induced β-cell failure (Navarro *et al.* 2018, Xu *et al.* 2019). Mishra *et al.* (2018) also observed elevated postprandial insulin secretion and decreased insulin sensitivity in rats following DHT treatment, while glucose-stimulated insulin secretion was exacerbated in islets cultured with DHT. This was accompanied by increased expression of rodent insulin genes *Ins1* and *Ins2*, likely regulated through the androgen response elements in their promoter regions (Mishra *et al.* 2018). Pancreatic abnormalities may also result from prenatal testosterone exposure. Sheep fetuses subjected to prenatal testosterone propionate had significantly elevated β-cell numbers, and elevated basal insulin secretion in adolescence, compared to controls (Ramaswamy *et al.* 2016). Similarly, prenatally testosterone-treated female sheep exhibited elevated fasting insulin and glucose-stimulated insulin hypersecretion at 5 weeks of age, without an evident difference in the rate of glucose disappearance after exogenous insulin administration (Recabarren *et al.* 2005). In addition, female non-human primates exposed to prenatal androgen excess demonstrate insulin hypersecretion after a glucose challenge in infancy (Abbott *et al.* 2010), and prenatal androgen administration also leads to the emergence of insulin resistance and β-cell dysfunction in female non-human primates during adulthood (Eisner *et al.* 2000), along with other PCOS-like traits (Abbott *et al.* 2009).

Circulating insulin levels reflect the rate of its metabolic clearance as well as the rate of its secretion, and both might contribute to hyperinsulinemia (Najjar & Perdomo 2019). Insulin clearance occurs primarily in the liver, where hepatocytes take up insulin through receptor-mediated endocytosis. Once in the hepatocyte, insulin degradation enzymes and acidic proteases within endosomes fully degrade the protein (Najjar & Perdomo 2019).

It seems that reduced insulin clearance is a significant contributor to hyperinsulinemia in PCOS. For instance, Tosi *et al.* (2020) found that insulin clearance was significantly reduced in two-thirds of women with PCOS, with impairments evident across all PCOS phenotypes, but most pronounced with increased obesity. O’Meara *et al.* (1993) observed elevated fasting insulin as a result of both reduced clearance and increased basal secretion in patients with functional ovarian hyperandrogenism compared to weight-matched controls. Ciampelli *et al.* (1997) found that in obese PCOS subjects, basal insulin clearance was reduced in the fasted state compared to controls, while all PCOS subjects showing a decreased insulin clearance rate in response to glucose, irrespective of BMI. More recently, Amato *et al.* (2015) found that while obese women with PCOS exhibited comparable insulin sensitivity to prediabetic controls in a hyperinsulinemic-euglycemic clamp, they had lower insulin clearance rates. Collectively, this suggests that diminished insulin clearance may be a fundamental feature that perpetuates hyperinsulinemia in some PCOS patients.

Reduced insulin clearance rates may be partially explained by elevated androgen levels. In obese PCOS subjects, baseline insulin clearance and T-lymphocyte insulin degradation were nearly halved compared to controls, and were negatively correlated with testosterone levels. Incubating non-PCOS control T-lymphocytes with testosterone *in vitro* also impaired insulin binding and degradation (Buffington & Kitabchi 1994). Tosi *et al.* (2020) observed an inverse relationship between serum androgen and insulin clearance rates, and found that androgen levels were independent predictors of insulin clearance rate but not of insulin secretion rate (Tosi *et al.* 2020).

### Hyperinsulinemia as an early PCOS feature that precedes insulin resistance

Elevated insulin levels without concurrent insulin resistance have been observed in lean PCOS patients and animal models. In a letrozole exposure mouse model of PCOS, insulin levels increased after 1 week of letrozole, despite no difference in insulin-induced glucose disposal at this time point. Instead, insulin resistance was observed after 5 weeks of letrozole treatment (Skarra *et al.* 2017). Body mass increased before its onset, so weight gain may have contributed to the development of insulin resistance in these mice (Skarra *et al.* 2017). Vrbíková *et al.* described lean PCOS subjects with higher early-phase glucose-stimulated insulin secretion than controls (Vrbíková *et al.* 2002), and found that lean PCOS subjects had elevated fasting insulin despite normal insulin sensitivity in hyperinsulinemic-euglycemic clamps, whereas obese women with PCOS were both insulin resistant and hyperinsulinemic (Vrbíková *et al.* 2004). Similarly, FSIVGTT testing revealed that nonobese PCOS patients had increased fasting and early-phase glucose-stimulated insulin levels than controls, without significant differences in fasting glucose or insulin sensitivity (Dumesic *et al.* 2016). Holte *et al.* (1994) also observed PCOS patients with glucose-induced insulin hypersecretion but no insulin resistance, which was more pronounced with increasing adiposity. Misra *et al.* (2024) observed significantly increased glucose-stimulated insulin secretion in lean PCOS women compared to BMI-matched controls, despite comparable insulin sensitivity in HOMA-IR and QUICKI indices.

It is difficult to disentangle causal relationships when insulin resistance is observed together with hyperinsulinemia, as is frequently the case with obese PCOS patients. However, the existence of disproportionate insulin hypersecretion without detectable insulin resistance in lean PCOS patients (who do not have obesity as a confounding factor) challenges the mechanism of compensatory hyperinsulinemia. Notably, hyperinsulinemia can also be detected in hyperandrogenic adolescent girls, despite normal glucose levels (Apter *et al.* 1995). Young PCOS patients also have higher fasting and/or glucose-stimulated insulin levels than non-PCOS controls who are matched for comparable degrees of insulin responsiveness, with significant correlations between fasting insulin and BMI regardless of PCOS status (Manco *et al.* 2014). Hyperinsulinemia may additionally be an early change for daughters of women with PCOS, whose insulin levels can be significantly elevated by late puberty compared to pubertal stage-matched controls (Kent *et al.* 2008).

Although the long-term effects of insulin exposure on PCOS pathogenesis have not been extensively studied, type 1 diabetes patients can provide some insights due to their requirement for exogenous insulin administration. PCOS is present in almost one of four women with type 1 diabetes, a significantly greater incidence than in the general population (Escobar-Morreale & Roldán-Martín 2016). PCOS and polycystic ovarian morphology are especially prevalent among women with intensive insulin treatment (three or more doses per day; Codner *et al.* 2006). This suggests that chronic insulin administration might predispose these patients to develop PCOS. However, insulin administration is not sufficient to induce PCOS-like phenotypes in animal models, so hyperinsulinemia is unlikely to be the sole cause of PCOS in the absence of other defects (Poretsky *et al.* 1988, Zhou *et al.* 2022).

Hyperinsulinemia contributes to PCOS pathogenesis by exacerbating hyperandrogenemia and other reproductive and metabolic dysfunctions. Despite the complex and bidirectional relationship between insulin sensitivity and insulin secretion, elevated insulin levels are often presented as a compensatory β-cell response to insulin resistance. However, hyperinsulinemia has been detected without concomitant insulin resistance in a subset of lean PCOS patients, hyperandrogenic adolescent girls, daughters of PCOS patients and early stages of PCOS animal models. Notably, androgens have the capacity to directly induce β-cell dysfunction and insulin hypersecretion. In addition, insulin clearance rates are often diminished in PCOS patients. Altogether, this lends support to an alternate model, in which hyperinsulinemia may act as an early defect in PCOS pathogenesis that precedes the impairment of insulin-induced glucose disposal, perhaps in a subset of the metabolically perturbed lean or obese patients who have been proposed to have a metabolic clinical onset of PCOS (Rotterdam phenotypes A-C; Table 1; Dapas *et al.* 2020, Unfer *et al.* 2024). This shifted metabolic framework points to the relevance of recognizing elevated insulin as a key PCOS feature even in the absence of aberrant glucose homeostasis, and highlights the importance of developing new and proactive management strategies to specifically prevent or suppress hyperinsulinemia rather than targeting insulin resistance or glucose levels (Nolan & Prentki 2019). Unfortunately, viable therapeutic approaches to directly limit β-cell insulin hypersecretion (rather than indirectly reducing it via lowering glucose levels) have not yet been established (Nolan & Prentki 2019).

## Insulin-sensitizing therapies in PCOS

Currently, PCOS symptoms are often managed through approaches that include lifestyle and diet interventions, combined oral contraceptives and insulin-sensitizing agents such as metformin (Al Wattar *et al.* 2021, Glendining & Campbell 2023). However, these treatments have varying efficacies depending on PCOS phenotypes and metabolic traits.

Metformin is the most common insulin-sensitizing agent used in PCOS. Metformin is a biguanide that mainly targets the liver, where it inhibits hepatocyte mitochondrial complex I, causing altered cellular energy levels that result in the activation of AMPK among other effects. AMPK decreases hepatic lipogenesis, increases fatty acid oxidation and decreases gluconeogenic gene expression, ultimately leading to improved insulin sensitivity and decreased hepatic gluconeogenesis, thus lowering insulin levels (Foretz *et al.* 2019). Of note, AMPK also directly decreases β-cell insulin secretion under hypoglycemic conditions (Szkudelski & Szkudelska 2019). Therefore, metformin might mitigate hyperinsulinemia to some degree through direct β-cell AMPK activation in addition to the indirect outcome that is secondary to its insulin-sensitizing role. Metformin also increases hepatic production of SHBG, and can directly inhibit theca cell androgen production, improving hyperandrogenism (Crave *et al.* 1995, Attia *et al.* 2001).

It appears that metformin might induce the greatest amelioration of PCOS symptoms and comorbidities in hyperinsulinemic, hyperandrogenemic patients. In obese PCOS patients, 6-month metformin treatment resulted in improved insulin sensitivity, menstrual regularity, and attenuated free testosterone and fasting insulin levels (Romualdi *et al.* 2011). Notably, metformin treatment had the greatest capacity to reduce insulin secretion, AMH levels and ovarian volume in the hyperinsulinemic group of obese PCOS patients compared to normoinsulinemic obese patients, in which metformin-improved insulin sensitivity and testosterone levels did not lead to these other changes (Romualdi *et al.* 2011). Among lean PCOS patients, metformin has varying impacts on parameters such as testosterone levels, insulin resistance, fasting insulin and menstrual regularity (Trolle *et al.* 2007). Önalan *et al.* (2005) found that metformin had the most pronounced effects on hirsutism and ovulatory regularity in lean hyperinsulinemic PCOS patients. Similarly, metformin had a greater capacity to reduce insulin, androgen and LH levels in those lean PCOS patients who were hyperinsulinemic based on their glucose-stimulated insulin secretion levels at baseline (Genazzani *et al.* 2007). This suggests that some of the reproductive and metabolic benefits of metformin may be partially due to its insulin-lowering effects in PCOS patients who have hyperinsulinemia.

Incretin-based therapies, particularly glucagon-like peptide-1 (GLP-1) receptor agonists, have been more recently indicated as promising insulin-sensitizing therapeutic options for PCOS. The gut-produced hormone GLP-1 facilitates postprandial glycemic control by augmenting glucose-dependent insulin secretion and suppressing glucagon secretion, while also slowing gastric emptying and promoting satiety (Nauck *et al.* 2021). In a meta-analysis comparing the efficacy of GLP-1 receptor agonists versus metformin in PCOS patients, GLP-1 receptor agonists were superior to metformin at improving HOMA-IR-based insulin sensitivity and lowering body mass index (Han *et al.* 2019). Similarly, in two mouse PCOS models, GLP-1 agonist-based therapies outperformed metformin at enhancing insulin sensitivity and lowering fasting insulin levels (Sánchez-Garrido *et al.* 2024). In addition to reducing body mass index, treatment with the GLP-1 receptor agonist liraglutide lowered serum testosterone levels in overweight and obese PCOS patients (Niafar *et al.* 2016), and has also been found to increase SHBG and improve menstrual regularity (Nylander *et al.* 2017). It remains to be determined whether GLP-1 receptor agonist therapies are more effective in patients with hyperinsulinemia, or to what extent the benefits are due to reducing body weight and ameliorating obesity-related comorbidities of PCOS. However, agents that enhance insulin sensitivity are typically associated with an accompanying reduction in insulin levels, highlighting the close relationship between these parameters. Moreover, depending on PCOS symptoms and subtype, these therapeutic agents can exert beneficial effects that extend beyond metabolic improvements.

## Conclusions

**Figure 3:**
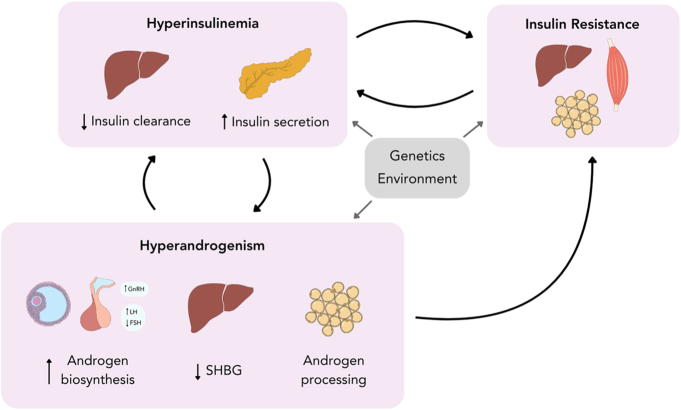
Alternative PCOS pathogenesis paradigm. In a subset of patients, hyperinsulinemia may be an early upstream defect that drives insulin resistance and exacerbates hyperandrogenism. Factors such as intrinsic genetic defects or a prenatal hyperandrogenic environment may lead to aberrant β-cell insulin secretion and hepatic insulin clearance, causing persistent hyperinsulinemia. Insulin can promote both ovarian and adrenal steroidogenesis, decrease production of SHBG and increase androgen production in adipose tissue, ultimately increasing levels of bioactive androgens. In turn, testosterone can act directly on β-cells to induce insulin hypersecretion, in addition to reducing hepatic insulin binding and degradation, collectively worsening hyperinsulinemia. Elevated insulin levels can downregulate insulin signaling by inducing receptor degradation and altering phosphorylation patterns of signal effector molecules, resulting in insulin resistance. Advanced stages of insulin resistance could cause compensatory hyperinsulinemia, further increasing insulin levels and its effects on the reproductive and metabolic symptoms of PCOS. A full color version of this figure is available at https://doi.org/10.1530/JOE-24-0269.

Evidence points to two non-mutually exclusive models of metabolic dysregulation in PCOS pathogenesis. Insulin resistance caused by obesity and/or insulin signaling aberrations may lead to compensatory hyperinsulinemia in some PCOS patients, consistent with the prevailing paradigm (Fig. 2). However, in a subset of individuals, hyperinsulinemia might be an early upstream defect that drives insulin resistance in addition to promoting hyperandrogenism and other features of PCOS (Fig. 3).

In this alternative framework, hyperinsulinemia could be caused by intrinsic defects in β-cells or insulin clearance mechanisms, or it may be a response to environmental conditions (such as pre- or post-natal exposure to hyperandrogenemia, inflammation or overnutrition) that stimulate increased β-cell mass, elevated insulin secretion, or reduced insulin degradation. High insulin levels may be a predisposing characteristic for other points of hormonal dysregulation through actions such as bolstering the hyperandrogenemia induced by genetic susceptibilities or environmental perturbations. In such a scenario, an elevation in androgens could further escalate hyperinsulinemia by impairing insulin clearance and inducing insulin hypersecretion. Hyperinsulinemia can lead to downregulated insulin signaling through means such as inducing receptor degradation, altering phosphorylation patterns of insulin signaling effector molecules (e.g., IRS-1, Akt, PI3K) in target tissues or indirectly by promoting obesity (Shanik *et al.* 2008, Nolan & Prentki 2019, Johnson 2021). Moreover, the resultant insulin resistance might perpetuate hyperinsulinemia by inducing even greater insulin secretion to accommodate for hyperglycemia. This would further exacerbate the impacts of insulin on ovarian steroidogenesis, hepatic SHBG production and androgen metabolism in peripheral tissues, thereby fueling the hyperandrogenemia-induced arrest of follicle development and oligo/anovulation.

Considering the multifactorial etiology and heterogeneous nature of PCOS, it is improbable that its metabolic characteristics always follow the same progression; there is unlikely to be a single, definitive paradigm. Since elevated insulin is a prevalent feature of PCOS that worsens hyperandrogenism and hypothalamic–pituitary–ovarian dysfunction, it is critical to investigate all of its potential causes, instead of presuming it is the consequence of peripheral insulin resistance. It may be particularly illuminating to examine the mechanistic causes of hyperinsulinemia that are associated with different PCOS subtypes, as its likelihood varies across phenotypes (Table 1; Moghetti *et al.* 2013). Elevated androgens have the capacity to directly and separately cause hyperinsulinemia and/or insulin resistance, and resolving the chronological progression of hyperandrogenism and hyperinsulinemia in PCOS is another ongoing challenge (Moghetti & Tosi 2021). Importantly, hyperinsulinemia can contribute to metabolic and reproductive disturbances even in the absence of detectable insulin resistance or altered glucose metabolism. It is crucial to acknowledge the multifaceted nature of hyperinsulinemia to fully understand and effectively manage PCOS disease progression.

## Declaration of interest

The authors declare that there is no conflict of interest that could be perceived as prejudicing the impartiality of this work.

## Funding

Research in the Templeman laboratory is supported by funding from the Canadian Institutes of Health Researchhttps://doi.org/10.13039/501100000024 (PJT-183618), the Natural Sciences Engineering Research Council of Canadahttps://doi.org/10.13039/501100000038 (RGPIN-2022-05149) and the Women’s Health Research Institute. EJH received a Canada Graduate Scholarship from the Canadian Institutes of Health Research and a British Columbia Graduate Scholarship. NMT is a Tier 2 Canada Research Chair in Cell Biology, a Michael Smith Health Research BC Scholar and a member of the Sexual and Reproductive Health and Rights Research Cluster at the University of Victoria.

## Author contribution statement

Both authors contributed toward conceptualizing and planning the review article and writing and critically revising the manuscript. EJH wrote the original draft and created the figures.
